# The Primary Alteration of Ventricular Myocardium Conduction: The Significant Determinant of Left Bundle Branch Block Pattern

**DOI:** 10.1155/2022/3438603

**Published:** 2022-12-20

**Authors:** Ljuba Bacharova, Bayes de Luna

**Affiliations:** ^1^International Laser Center CVTI, Bratislava, Slovakia; ^2^Cardiovascular Research Foundation, Cardiovascular ICCC-Program, Research Institute Hospital de la Santa Creu i Sant Pau, Barcelona, Spain

## Abstract

Intraventricular conduction disturbances (IVCD) are currently generally accepted as ECG diagnostic categories. They are characterized by defined QRS complex patterns that reflect the abnormalities in the intraventricular sequence of activation that can be caused by pathology in the His-Purkinje conduction system (HP) or ventricular myocardium. However, the current understanding of the IVCD's underlying mechanism is mostly attributed to HP structural or functional alterations. The involvement of the working ventricular myocardium is only marginally mentioned or not considered. This opinion paper is focused on the alterations of the ventricular working myocardium leading to the most frequent IVCD pattern—the left bundle branch block pattern (LBBB). Recognizing the underlying mechanisms of the LBBB patterns and the involvement of the ventricular working myocardium is of utmost clinical importance, considering a patient's prognosis and indication for cardiac resynchronization therapy.

## 1. Introduction

The ECG diagnostic classification of intraventricular conduction disturbances (IVCD) is currently generally well-defined and accepted and represents a standard diagnostic ECG classification. They refer to abnormalities in the ventricular activation sequence, and, as stated in the scientific statement from the AHA/ACCF/HRS recommendations, “they may be caused by structural abnormalities in the His-Purkinje conduction system or ventricular myocardium that result from necrosis, fibrosis, calcification, infiltrative lesions, or impaired vascular supply” [1].

The His-Purkinje conduction system in the ventricles represents well defined and visible structures, and its role in ventricular impulse propagation was early recognized. After some confusion [2], the characteristic QRS patterns corresponding to specific blocks in the conduction system have been defined and related to individual anatomical parts of the conduction system, defining the bundle branch and fascicular blocks, respectively. Logically, it is assumed that if the impulse propagation in the conduction system is blocked, the ventricular activation in the corresponding myocardial region is delayed and results in the typical QRS patterns.

Although the participation of the ventricular working myocardium in the intraventricular conduction disturbances is explicitly mentioned in the AHA/ACCF/HRS recommendations [1], the participation of the pathologically changed myocardium in altered ventricular activation is somehow vague. It is not sufficiently elucidated and/or explicitly defined, both in the bundle branch blocks as well as in the fascicular blocks.

In this opinion paper, we stress the role of the pathologically changed ventricular myocardium itself in affecting the sequence of electrical impulse propagation and consequently resulting in the typical QRS patterns. It is demonstrated by using the left bundle branch block (LBBB) as an example.

## 2. Intraventricular Conduction Alterations in the Structural Heart Diseases: The Association of LBBB Pattern with Cardiac Pathology

It has been repeatedly documented that the majority of LBBB patients have an underlying heart disease, leading to structural changes of the left ventricular myocardium, the most common being LV hypertrophy/dilation, fibrosis, ischemic heart disease, and cardiomyopathies, as well as advanced age [3–7]. Additionally, there are other less frequent conditions that can be associated with LBBB, such as acute rheumatic carditis, Wegener granulomatosis, cardiac involvement of metastatic breast cancer, bacterial endocarditis, sarcoidosis, S/P chest radiotherapy, and quadriplegia with syringomyelia postspinal cord injury [8]. It was also shown that 89% patients with LBBB develop a significant cardiovascular disease later [5]. Here we describe the major factors causing slowed ventricular activation propagation in selected cardiac conditions with the most frequent occurrence of LBBB: LVH, ischemia, cardiomyopathy, and advanced aging.

### 2.1. Left Ventricular Hypertrophy

In clinical practice, the LVH definition is reduced to the increase in LV size/mass—in this respect, the imaging methods are superior for the LVH diagnosis. It needs to be stressed, however, that the increase in the left ventricular size/left ventricular mass (LVM) is associated with a complex interrelated structural and functional rebuilding of the ventricular myocardium, considerably affecting active and passive electrical properties, and consequently the sequence of ventricular activation.

Hypertrophied cardiomyocytes differ from normal cardiomyocytes in diameter and length, as well as in branching and the number of connected cardiomyocytes [9–11]. At the molecular level, a variety of alterations in ion channels, gap junctions, and connexin43, i.e., in the dominant molecular processes affecting electrogenesis, have been documented [12–15]. It includes changes in the upstroke velocity of the action potential, the action potential propagation, the conduction velocity, and the cell-to-cell impulse propagation [16–18]. The conduction velocity of individual hypertrophied cardiomyocytes is affected [15, 19], as is the intercellular coupling. The density, distribution, and organization of the gap junctions (GJ), crucial for uninterrupted propagation of electrical impulse in the LVH, are significantly altered [20, 21].

Hypertrophic rebuilding also includes changes in the interstitium, including fibrosis, inflammation, degenerative changes, and apoptosis [22]. The fibrosis can be diffused as well as it can create localized fibrous tissue areas, e.g., the mid-wall fibrosis described in patients with LVH [23, 24]. The inflammation also creates localized areas of edema and accumulated blood cells [25].

All of these changes can demonstrate considerable heterogeneity, including the size of hypertrophic cardiomyocytes [26, 27], the membrane currents [28], the connexin expression, and the action potential characteristics [29]. Diffused and localized areas of pathologically changed myocardium thus contribute to the slowing and heterogeneity of the electrical impulse propagation in the ventricles.

### 2.2. Ischemia

The altered conduction in the ischemic myocardium is studied extensively as a factor creating conditions for developing arrhythmias [30]. Ischemia affects both the action potential duration and morphology as well as the conduction velocity. This effect can be regional, as it is, e.g., in the coronary heart disease, or more diffuse as it is, e.g., in hypertrophy. The conduction alterations are present in areas of acute as well as chronic ischemia and/or scattered fibrosis [31–33].

Acute ischemia affects the morphology of the action potential [34, 35] and the ventricular activation is slowed [36, 37], consequently, the ventricular activation pattern is changed as it has been documented in animal as well as in human studies [32, 36, 38–41].

Recent findings of animal and human studies using advanced technologies are consistent with those previous findings [30, 42, 43]. Also, the AHA/ACCF/HRS recommendations consensus report on intraventricular conduction disturbances [1] recognizes ischemia-related slowed conduction, using the terms “possible peri-infarction block,” characterized by the changes in QRS, and “peri-ischemic block,” defined as a transient increase in QRS duration accompanying the ST segment deviation observed in acute injury.

### 2.3. Cardiomyopathy

Cardiomyopathy is a rather general term for a whole spectrum of myocardium diseases of ischemic and nonischemic origins affecting cardiomyocytes as well as interstitium. It has been shown that the probability of cardiomyopathy in LBBB patients is high [44], and the occurrence of LBBB is more frequent compared to the right bundle branch block [45].

In cardiomyopathy both the myocytes, as well as the interstitium are considerably affected. In general, histological examination shows hypertrophied cardiomyocytes/prolonged cardiomyocytes with bizarre shapes and pleiotropic nuclei; the regular myocardial architecture is disarrayed [46–50], which naturally affects the activation propagation. The important factor contributing to the altered activation sequence is the disorganization of the intercalated discs [51, 52].

A common characteristic of cardiomyopathies is the increased proportion of interstitial fibrosis [46–48], which can be currently well quantified by CMR late gadolinium enhancement (LGE) [30]. Changes of cardiomyocytes together with the fibrosis result in a slow and heterogeneous impulse propagation, creating substrate for initiating and maintaining ventricular arrhythmias [53–55]. Naturally, the QRS morphology reflects the altered sequence of depolarization.

### 2.4. Advanced Aging

The incidence of LBBB is progressively increasing with age [5]. It is documented that about 6% of 80-year-old individuals have LBBB, compared to 1% of people younger than 50 years [56].

Aging myocardium undergoes substantial changes affecting the ventricular impulse propagation. There are changes in the conduction system, but the working myocardium is also considerably affected. These changes include progressive cardiomyocyte hypertrophy, inflammation, and the gradual development of cardiac fibrosis [57, 58].

These examples of cardiac pathology demonstrate the substantial changes having impact on the conduction velocity in the ventricular myocardium and consequently on the QRS morphology.

## 3. Computer Simulations: The Effect of the Regional and Diffuse Conduction Slowing in the Working Myocardium on the Resultant QRS Pattern

It is complicated to estimate the effect of the slowed conduction and/or the block separately in the conduction system or in the working myocardium on the resultant QRS patterns in the clinical setting. However, it can be addressed by using computer simulations.

**Figure 1:**
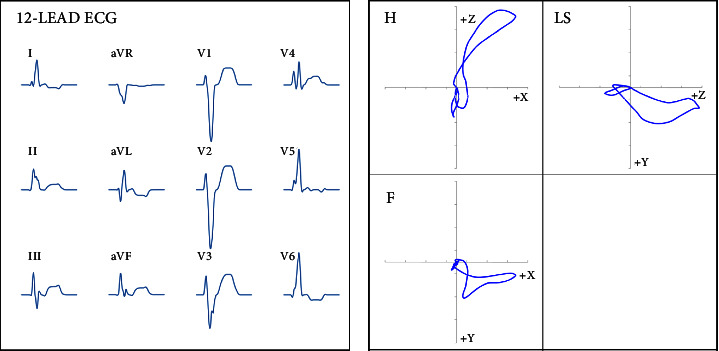
Results of the computer simulation: Typical LBBB patterns observed after slowing the conduction velocity in left ventricular myocardium without blocking the onset of left ventricular activation, i.e. without simulating the block in the left bundle branch. 12-lead ECG (a), vectorcardiogram (b), (H) horizontal plane, LS: left sagittal plane, (F) frontal plane. Adapted from: Bacharova L, Szathmary V, Mateasik A. Electrocardiographic patterns of left bundle-branch block caused by intraventricular conduction impairment in working myocardium: a model study. J Electrocardiol. 2011; 44 : 768–78. With permission.

We addressed this issue using two different computer models [59, 60]. Typical LBBB patterns were observed both when blocking or delaying the onset of left ventricular activation as well as when slowing the conduction velocity in the working ventricular myocardium [61–63] or when simulating the uncoupling in the working myocardium [61]. The results of these simulations are consistent with the ECG criteria for LBBB including QRS duration, QRS complex morphology, and *T* wave orientation (Figure 1).

The assumption of the involvement of the working myocardium in the LBBB patterns is not new [4, 64, 65]. Although the slowed conduction velocity in ventricular myocardium is mentioned also in the AHA/ACCF/HRS recommendations [1] as a possible reason for IVCD, the slowed conduction in the ventricular myocardium is not primarily explicitly related to LBBB.

Explicitly in relation to LBBB patterns, Surawicz and Knilans [66] assume that QRS complex duration >150 ms indicates myocardial structural or functional abnormalities causing additional delay of the ventricular activation in LBBB patients. On the other hand, Strauss et al. [67] defines the combination of prolonged QRS duration ≥140 ms for men and ≥130 ms for women (associated with mid-QRS notching or slurring in ≥2 contiguous leads as the “true” LBBB and he assumes that the LBBB patterns with shorter QRS duration could be caused probably by a combination of LVH and left anterior fascicular block. Although these two statements are opposed, there is an important message in both—the consideration of the altered conduction in the working myocardium in the LBBB pattern interpretation. This message is important in the context of the electrophysiological findings that even in the complete LBBB pattern the conduction velocity in the left bundle can be normal or only slowed [68–70].

Using the term “left bundle branch block,” the dominant message is the impaired conduction in the left bundle branch. The involvement of the pathologically changed ventricular myocardium in the ventricular depolarization slowing is not verbalized, underestimated, or even neglected. However, recognizing the degree of myocardial impairment and resultant slowing of ventricular activation is of the utmost importance in evaluating patients' status. Slowed conduction velocity creates conditions for re-entry and is recognized as the arrhythmogenic substrate correlating with ventricular arrhythmias and sudden cardiac death. As well, knowledge of the degree of myocardial impairment can be key for indicating patients for resynchronization therapy. There is still a high number of “nonresponders” [71]. If the myocardium is severely impaired, then the effect of the electrical stimulation should be obviously different in patient with less or only slightly impaired myocardium.

Summarizing, the LBBB patterns reflect the slowed activation sequence of the left ventricle that can be caused by: (1) the electrical impulse block/slowing in the left bundle branch; (2) primarily slowed activation in the left ventricular myocardium; and (3) a combination of both. The sole affection of LBB (Lenegre disease) [72] is reported only in minority of patients with the LBBB pattern, and even in these patients, the myocardium can be affected. The odds of having just an isolated alteration of LBB are therefore low, and the alteration of myocardium is highly probable.

The LBBB is presented here as an example. Analogically, the involvement of impaired ventricular myocardium also needs to be also considered in other intraventricular conduction disturbances where the terminology and pathophysiological interpretation imply impairment in the H-P conduction system, such as right bundle branch block, and fascicular blocks. In cases of fascicular block the same questions need to be answered—is it the isolated impairment of the anterior, posterior, and middle fascicles, analogically to the Lenegre [72] or Lev diseases [73], or is it associated with or caused by the myocardial impairment? Or, can myocardial impairment itself result in an ECG pattern of fascicular block? In the simulation study [63], we showed that changed electrical properties of the myocardium, namely, the transmural conduction slowing in anteroseptal or posterior locations, resp., resulted in QRS complex changes suggestive of fascicular blocks (anterior and posterior, resp.). This assumption can be supported by the results of the clinical studies [74–76].

It also needs to be mentioned, however, that patients with severe LV structural alterations and clinical heart failure symptoms can develop a pattern of the right bundle branch block (RBBB). In these patients, the sequence of ventricular activation differs as compared to patients with the LBBB pattern [67, 73, 74]: the ventricular activation reaches the posterior and lateral walls before the anterior septum and anterior wall are activated, as opposed to patients with LBBB patterns, where the last activated regions are the inferoposterior and lateral walls. This clinical condition is associated with a higher in-hospital arrhythmic risk and mortality and a worse prognosis after discharge [67, 73, 75]. These findings further emphasize the importance of understanding the interrelationship of structural damage to the myocardium, activation sequence, and resultant QRS patterns.

In cases of fascicular block, the same questions need to be answered—is it the isolated impairment of the anterior, posterior, and middle fascicles, analogically to the Lenegre [72] or Lev diseases [76], or is it associated with or due to myocardial impairment? Or, can myocardial impairment itself result in an ECG pattern of fascicular block? In the simulation study [63], we showed that changed electrical properties of the myocardium, namely, the transmural conduction slowing in anteroseptal or posterior locations, resp., resulted in QRS complex changes suggestive of fascicular blocks (anterior and posterior, resp.). This assumption is also supported by the results of the clinical studies [32, 77, 78].

Moreover, it has been shown in a canine model of acute ischemia as well as in patients with acute myocardial infarction that the working ventricular myocardium is less resistant to ischemia compared to the conduction system [33, 79]. Therefore, the assumption on the essential contribution of the primary impairment of working myocardium in slowing the ventricular activation is logical.

## 4. Conclusion

**Figure 2:**
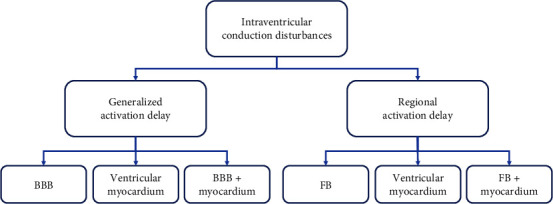
In the intraventricular conduction disturbances the activation of the whole ventricle or its part is delayed. In the generalized intraventricular block the delay can be caused by: (1) The delayed onset of activation due to blocks in H-P system; (2) slowed activation propagation in the pathologically changed ventricular myocardium; (3) combination of both causes. In the regional intraventricular block the delay can be caused by: (1) The delayed onset of activation in particular ventricular area due to blocks in fascicles, (2) slowed activation propagation in the particular ventricular region due to locally pathologically changed myocardium, (3) combination of both causes. BBB: bundle branch blocks, FB: fascicular blocks.

Considering the electrophysiological background, the LBBB pattern can result from (Figure 2):

- The block/slowing of the impulse propagation in the left bundle branch and the consequent delay of LV activation;
- The primarily slowed ventricular activation in the working left ventricular myocardium due to its structural and/or functional alteration;
- The combination of the two above, i.e., the combination of the left bundle branch block and primarily slowed left ventricular activation.

These categories are in accordance with the recent approach in resynchronization therapy using the His bundle pacing or the left bundle pacing in patients with “true” LBBB. Several studies show improved clinical outcomes compared to biventricular pacing [80, 81]. However, according to the results of the systematic review, their benefit needs to be verified by randomized controlled studies [82]. Considering the persisting number of patients not benefiting from CMR, the location of the lead positions remains challenging with respect to the specific left ventricular asynchrony patterns [83].

There is time to reconsider the traditional ECG classification and interpretation and to link them to the current knowledge of structural and electrophysiological characteristics of the heart in the cardiac pathology. The suggested interpretation of LBBB corresponds better with the pathophysiological understanding of the intraventricular conduction disturbances and understanding of the underlying etiological factors. The LBBB, as well as other ventricular conduction blocks, should be identified as markers of ventricular electrical/structural remodeling of the myocardium, with diagnostic and prognostic implications, such as risk of arrhythmia, treatment of heart failure, indication for resynchronization therapy, and pacing.

## Data Availability

Data sharing is not applicable to this article as no datasets were generated or analysed during the current study.
